# Analysis of Macrolide Resistance in *Bordetella pertussis* Isolated from Japanese Children in 2025 Using Test Kit and Sequence Method

**DOI:** 10.3390/biomedicines14010167

**Published:** 2026-01-13

**Authors:** Tomohiro Oishi, Takashi Nakano

**Affiliations:** 1 Department of Clinical Infectious Diseases, Kawasaki Medical School, 577, Matsushima, Kurashiki 701-0192, Okayama, Japan; 2 Department of Pediatrics, Kawasaki Medical School, 577, Matsushima, Kurashiki 701-0192, Okayama, Japan; nakano@med.kawasaki-m.ac.jp

**Keywords:** *Bordetella pertussis*, macrolide resistance, sequencing, pediatric infections, gene amplification

## Abstract

**Background**: *Bordetella pertussis* causes pertussis, a respiratory infection with whooping cough. Despite a high vaccine coverage, pertussis resurged post-COVID-19 pandemic. Meanwhile, isolates resistant to macrolides—the first-line therapy—have increased in several countries, including Japan. Culturing *B. pertussis* and detecting resistance are difficult; reports remain limited in Japan. **Methods**: From March to August 2025, we collected nasopharyngeal samples from children aged 0–15 years with suspected pertussis at six Japanese clinics. Pediatricians obtained swabs and tested them using gene-amplification kits (e.g., BioFire^®^ SpotFire^®^ in four clinics, LAMP Pertussis Detection^®^ in two clinics). *B. pertussis* was confirmed by PCR; isolates were sequenced to identify macrolide-resistant mutations. **Results**: Samples were taken from 54 children, the number of boys and girls was 34 and 20, and their median age was 12 years old. Among 54 *B. pertussis* isolates, 43/52 (82.7%) sequenced strains harbored the A2047G mutation associated with macrolide resistance. Resistance rates at each clinic varied from 40% to 96%. **Conclusions:** These findings indicate a post-pandemic rise in macrolide-resistant *B. pertussis* in Japan. Ongoing resistance surveillance is essential, and repurposing residual clinical samples after routine testing is useful given culture and detection challenges.

## 1. Introduction

Pertussis, caused by *Bordetella pertussis*, is a respiratory infection typically accompanied by whooping cough. This can present as a severe, life-threatening illness, especially among young infants. Therefore, vaccination against *B. pertussis* is widespread worldwide; the World Health Organization reported that 85% received three doses of the diphtheria–pertussis–tetanus (DPT) vaccine in 2024 [1].

However, immunity against pertussis is waning and is reported to persist for only 4–12 years [2,3,4,5,6]. Therefore, pertussis affects not only infants without vaccination but also adolescents a long time after the last vaccination. In fact, endemics of pertussis have recently arisen mainly among schoolchildren who have lost immunity despite past vaccination [7].

During the coronavirus disease [COVID-19] pandemic, pertussis, like other respiratory infections, was under control, but it resurged after the COVID-19 pandemic in many countries [8,9]. Furthermore, isolates that are resistant to the first-line drug, macrolide antibiotics, have been increasing, especially in China [10]. In Japan, a patient with macrolide-resistant *B. pertussis* that was detected in 2018 was first reported in 2020 [11]. After the COVID-19 pandemic, pertussis has increased rapidly up to 2025 [12]. Moreover, during the COVID-19 era, the misuse and overuse of unspecific antibiotics led to an increase in bacterial resistance to antibiotics [13,14]. This also might be influenced by pertussis; reports on macrolide-resistant *B. pertussis* have been increasing [15]. However, there have been no epidemiologic reports on the resistance rate to macrolide among *B. pertussis* isolates in Japan. In general, antimicrobial susceptibility testing is needed to determine resistance to antibiotics by culture, but *B. pertussis* is difficult to grow and identify by culture, especially from clinical specimens [16]. The mechanism of macrolide resistance is known to be an A2047G mutation of the 23S rRNA gene, which is connected with macrolide agents [17]. Therefore, several polymerase chain reaction [PCR] methods have been reported to detect macrolide-resistant *B. pertussis* recently [18,19]. Furthermore, gene-amplification kits able to detect *B. pertussis* in a short time using simple machines are currently available [20,21]. Therefore, we investigated the rate of macrolide-resistant *B. pertussis* in Japan by using PCR among *B. pertussis* isolates detected by certain gene-amplification kits.

## 2. Materials and Methods

### 2.1. Materials

We collected *B. pertussis* (BP) samples from March to August 2025 from the nasopharyngeal specimens of children ages 0 to 15 years, which is the age group for children for pediatrics in Japan, with suspected pertussis due to BP. The cooperating medical settings consisted of 6 facilities—all clinics—located in the Chubu, Kinki, and Chugoku regions of Japan. We enrolled the children of outpatients and inpatients under 15 years of age. Pediatricians who cooperated with our research collected nasopharyngeal swabs from children at their facilities. They sent their nasopharyngeal swab specimens using courier services to our laboratory at Kawasaki Medical School at room temperature within 2 days.

### 2.2. Real-Time PCR Analysis for Detection of B. pertussis

Samples were collected as follows. Pediatricians obtained nasopharyngeal samples from children suspected of having *Bordetella* infection using sterile swabs (JCB Industry Limited, Tokyo, Japan). The samples were then examined for *B. pertussis* using test kits, either BioFire^®^ SpotFire^®^ (Biomerieux, Marcy l’Etoile, France) or the Loop-Mediated Isothermal Amplification Method^®^ (LAMP Pertussis Detection^®^, Eiken, Tokyo, Japan). Thereafter, samples were sent to our laboratory if *B. pertussis* was detected. Specifically, samples were placed into 3 mL of universal viral transport medium (Becton, Dickinson and Company, Sparks, MD, USA) and transported at room temperature within 2 days to our laboratory.

We extracted crude DNA as follows: First, 300 mL of suspended transport medium was transferred into a 1.5 mL microtube. Next, it was centrifuged at 4 °C at 20,000× *g* for 30 min. Then, 285 mL of the supernatant was removed, and the remains were transferred into a thin-walled 200 mL PCR tube which included 85 mL of lysis buffer [2 mmol/L Tris-HCl (pH 8.3), 10 mmol/L of KCl, 0.045 mmol/L of MgCl_2_, 0.45% Triton X-100, 0.45% Tween 20, and 0.4 mg/mL of RNA-grade Proteinase K (Thermo Fisher Scientific Inc., Waltham, MA, USA)]. Finally, this suspension was pipetted gently to mix, and the PCR amplifier was set as follows: incubation at 55 °C for 60 min, incubation at 100 °C for 10 min, and cool to 4 °C.

### 2.3. Detection of Mutations Related to Macrolide Resistance Among B. pertussis Isolates

We referred to past studies for PCR-based sequencing methods for directly detecting mutations related to macrolide resistance among *B. pertussis* [19].

In particular, we designed primers based on the sequence of domain V of the 23S rRNA gene of the Chinese *B. pertussis* vaccine strain CS (GenBank accession number CP002695.1). As shown in Table 1, the sequences of the primers were 1505F: GGCACGAGCGAGCAAGTCTC and 2118R: TCTGGCGACTCGAGTTCTGC.

We prepared the PCR reaction mixture as follows. DNase/RNase-Free Distilled Water (18.0 μL), PrimeSTAR^®^ HS (Takara, Kusatsu, Japan) (25.0 μL), Primer_1505F (5 pmol/μL) (1.0 μL), Primer_2118R (5 pmol/μL) (1.0 μL), and Template DNA 5.0 μL were used to prepare a total of 50.0 μL of PCR reaction mixture.

The DNA fragments were generated by 5 cycles of 10 s at 98 °C, 30 s at 68/66/64/62/60 °C (decreasing each cycle), and 45 s at 68 °C, followed by 10 cycles of 10 s at 98 °C, 30 s at 58 °C, and 45 s at 68 °C. Final extension was 7 min at 68 °C.

The PCR products were sent to Eurofins Co., Ltd. for direct sequencing analysis, which determined the nucleotides at positions 2047 in domain V of the 23S rRNA gene, which are associated with resistance to macrolides.

### 2.4. Ethical Statement

The study protocol was approved by the Ethics Committee of Kawasaki Medical School, Kurashiki, Japan, on 14 December 2024 (no. 3119-08). We followed the Declaration of Helsinki in our study.

## 3. Results

### 3.1. Background of B. pertussis Isolates

**Figure 1:**
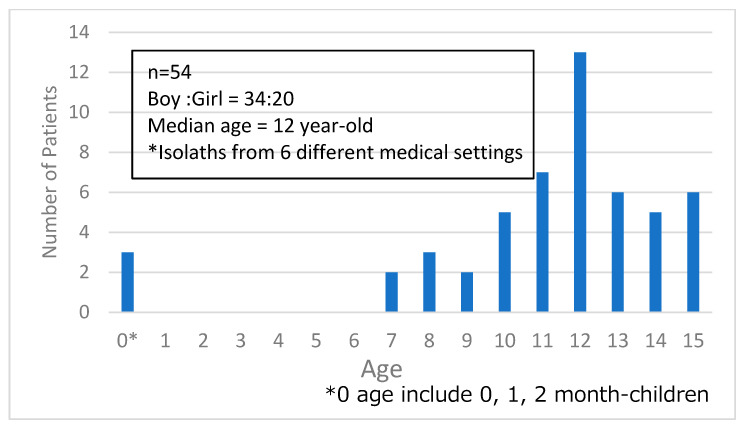
Age distribution of children in whom *B. pertussis* isolates were detected.

During the study period, a total of 52 *B. pertussis* isolates from Japanese children were collected and analyzed. The male-to-female ratio and median age of the originating patients were 32:20 and 12 years, respectively. Most patients were ≥7 years old, apart from three infants who were <1 year old (Figure 1).

### 3.2. Distribution of Macrolide-Resistant B. pertussis Due to a Mutation in the 23S rRNA Gene Isolated from Japanese Children in 2025

**Figure 2:**
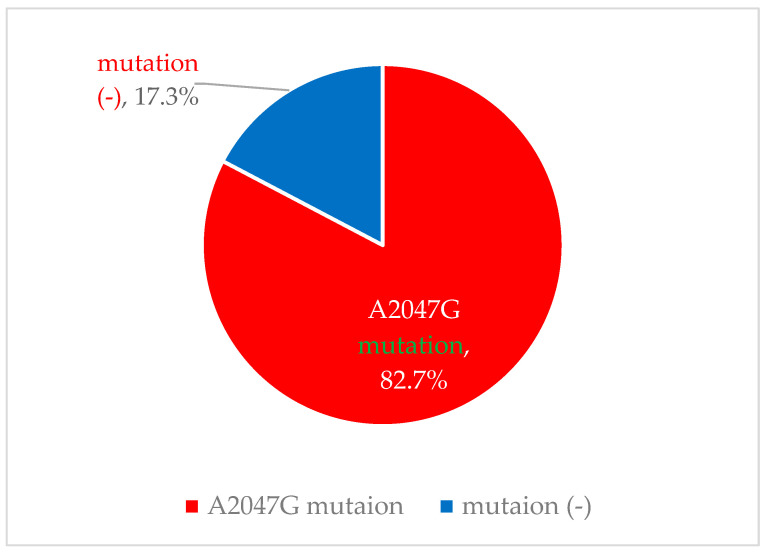
Mutations among *B. pertussis* isolates (n = 54).

Among 52 *B. pertussis* isolates, 82.7% (43/52) harbored the A2047G mutation, and 17.3% (9/52) had no mutation (Figure 2). The backgrounds of the originating children and the distribution of macrolide-resistant *B. pertussis* rates in each medical setting are shown in Table 2. For the test kits for *B. pertussis*, SpotFire^®^ was used in four clinics, and LAMP pertussis detection^®^ in two. Of these, there were only a few enrolled children in two medical settings. In the other four settings, the rates of macrolide-resistant *B. pertussis* ranged from 40.0% to 96.0%.

## 4. Discussion

In our study, we used samples that had been tested by two different methods to detect *B. pertussis* before transport. The sensitivity and specificity of these kits for *B. pertussis* are reportedly 71.4% and 100.0% compared to real-time PCR in LAMP [22], but those for only *B. pertussis* for SpotFire^®^ are not clear. Therefore, we cannot deny that some children with pertussis escaped detection.

*Bordetella parapertussis* (*B. parapertussis*), which is rarely isolated in practice [23], is closely related to *B. pertussis* and can also cause pertussis-like coughs. However, both methods can distinguish *B. pertussis* from *B. parapertussis* [20,21]. Therefore, all isolates in our study were confirmed as *B. pertussis*, not *B. parapertussis.*

Most enrolled children were ≥7 years old (i.e., schoolchildren). This age group most often presented with pertussis in the 2025 outbreak in Japan [24]. Thus, the age trend in our study likely reflects the national pattern. In contrast, in the United States, the age group most often affected is <1 year old, not schoolchildren [8]. This difference is attributed to national immunization schedules. Specifically, vaccination against pertussis in the United States is administered four times by 18 months and boosted at 4–6 years and 11–12 years [25]. In Japan, vaccination is not performed after the initial four doses under the national program [26]. Correspondingly, antibody levels against the pertussis toxin among healthy Japanese children decrease from 6–11 months to 5–9 years [27].

The rate of macrolide resistance among *B. pertussis* isolates was high (>80%). As noted, there have been no reports since macrolide resistance among *B. pertussis* isolates was first reported in Japan in 2018 [11]. This reason for this is not clear, but it is possible that *B. pertussis* isolates and the macrolide resistance among them were unable to be detected because no items such as gene-amplification kits were used in our research then. Macrolide-resistant *B. pertussis* already existed even before the COVID-19 pandemic [26]. In China, the rate of macrolide resistance among *B. pertussis* was already reported to be 85.2% in 2017–2019. The reason for the increase in macrolide-resistant *B. pertussis* strains in China is unclear. Furthermore, macrolides are recommended as the first-choice therapy for *B. pertussis* infections, including in Japan [28]. At present, bedside testing to detect macrolide-resistant *B. pertussis* is not available. Consequently, many patients with *B. pertussis* may receive macrolides, and some may improve clinically despite bacterial persistence, potentially transmitting these isolates to others. Thus, macrolide-resistant *B. pertussis* may have spread rapidly in Japan.

We enrolled six clinics, and there was little difference in the rate of macrolide-resistant *B. pertussis* among them. The reason for the variation was unclear, but it might reflect differences in how often macrolide-resistant *B. pertussis* flowed in from outside the region.

There were some limitations in our study.

First, the clinical courses of patients from whom *B. pertussis* was isolated were not analyzed. As noted, some patients with macrolide-resistant *B. pertussis* improved symptomatically despite receiving macrolides [29]; therefore, some children in our cohort who were treated with macrolides but had macrolide-resistant *B. pertussis* isolates might also have improved. Furthermore, we also need to research co-infection to study the clinical courses. In our study, some children used SpotFire^®^ and multiplex PCR, and there were no isolated pathogens other than *B. pertussis*. We plan to analyze clinical outcomes in the future.

Second, we only detected the A2047G mutation in domain V of the 23S rRNA gene and did not analyze other loci or perform drug-susceptibility testing to antibiotics beyond macrolides. Regarding the former, A2047G is the only point mutation isolated in association with macrolide resistance in *B. pertussis* [18]. However, the detection of resistance in the gene may not 100% indicate resistance. Therefore, culture is required for antibiotic susceptibility testing. However, as described above, our samples were transported in universal viral transport medium, which includes vancomycin and polymyxin B to prevent contamination [30]. Among these, polymyxin B might affect *B. pertussis*, making culture infeasible. Furthermore, recently, new antibiotics for drug-resistant Gram-negative bacteria have been developed, such as the antibiotics targeting bacterial metallophores and siderophore molecules [31,32]. Therefore, we plan to use alternative transport media which do not include any antibiotics to increase the detection of *B. pertussis* for culture and investigate the susceptibilities of antibiotics other than macrolides in future work.

## 5. Conclusions

We investigated macrolide resistance among *B. pertussis*, which resurged post-COVID-19 pandemic in 2025. The *B. pertussis* samples were collected from nasopharyngeal samples from children using gene-amplification kits, and were sequenced to identify macrolide-resistant mutations, i.e., the A2047G mutation in domain V of the 23S rRNA gene.

It was determined that the rate of macrolide resistance among *B. pertussis* isolates exceeded 80%, but there was little difference in the rate across regions. Thus, macrolide resistance was observed in the majority of *B. pertussis* isolated in Japan. Therefore, future studies should include more isolates and assess susceptibilities to antimicrobial agents other than macrolides in the case of infection caused by *B. pertussis*.

## Figures and Tables

**Table 1:** Primer pair sequences for detecting mutations associated with macrolide resistance among *B. pertussis* isolates from nasopharyngeal samples.

Primer	Primer Sequence (5′ to 3′)	Product Size
1505F	GGCACGAGCGAGCAAGTCTC	614 bp
2118R	TCTGGCGACTCGAGTTCTGC	

**Table 2:** Backgrounds of the originating children and distribution of macrolide-resistant *B. pertussis* due to the A2047G mutation in 23S ribosomal RNA across medical settings.

Medical Setting (Region/Hospital or Clinic)	Test Kit	Total Number	Boys: Girls	Age of Children	Distribution of Macrolide-Resistant *B. pertussis*
A (Chubu/Clinic)	SpotFire^®^	9	3:6	7–15	88.9% (8/9)
B (Chubu/Clinic)	SpotFire^®^	5	4:1	0–14	40.0% (2/5)
C (Chubu/Clinic)	SpotFire^®^	25	18:7	8–14	96.0% (24/25)
D (Kinki/Clinic)	SpotFire^®^	1	1:0	0	100% (1/1)
E (Chugoku/Clinic)	LAMP^®^	11	5:6	0–15	63.6% (7/11)
F (Chugoku/Clinic)	LAMP^®^	1	1:0	0	100% (1/1)
Total	SpotFire^®^: 4 LAMP^®^: 2	52	32:20	Median: 12 (0–15)	82.7% (43/52)

## Data Availability

The raw data supporting the conclusions of this article will be made available by the corresponding author, Tomohiro Oishi, on request.

## Abbreviations

The following abbreviations are used in this manuscript:

BP	Bordetella Pertusis
DPT	Diphtheria–Pertussis–Tetanus vaccine
PCR	Polymerase Chain Reaction
WHO	World Health Organization
